# METTL3 promotes colorectal cancer progression through activating JAK1/STAT3 signaling pathway

**DOI:** 10.1038/s41419-023-06287-w

**Published:** 2023-11-25

**Authors:** Yuechao Sun, Weipeng Gong, Song Zhang

**Affiliations:** 1 Ningbo Institute of Life and Health Industry, Chinese Academy of Sciences, Ningbo, Zhejiang The People’s Republic of China; 2 Department of Gastrointestinal Surgery, Shandong Cancer Hospital and Institute, Shandong First Medical University and Shandong Academy of Medical Sciences, Jinan, Shandong The People’s Republic of China; 3 Shandong Provincial Key Laboratory of Radiation Oncology, Shandong Cancer Hospital and Institute, Shandong First Medical University Affiliated Tumor Hospital, Jinan, Shandong The People’s Republic of China

**Keywords:** Colon cancer, RNA

## Abstract

The role of METTL3-mediated N6-methyladenosine (m^6^A) modification has been elucidated in several cancers, but the concrete mechanism underlying its function in colorectal cancer is still obscure. Here, we revealed that upregulated methyltransferase-like 3 (METTL3) in colorectal cancer exerted both methyltransferase activity-dependent and -independent functions in gene regulation. METTL3 deposited m^6^A on the 3’ untranslated region of the JAK1 transcript to promote JAK1 translation relying on YTHDF1 recognition. Besides, METTL3 was redistributed to the STAT3 promoter and worked in concert with NF-κB to facilitate STAT3 transcription, which was achieved independently on METTL3 methyltransferase activity. The increased JAK1 and STAT3 corporately contributed to the activation of the p-STAT3 signaling pathway and further upregulated downstream effectors expressions, including VEGFA and CCND1, which finally resulted in enhanced cancer cell proliferation and metastasis in vitro and in vivo. Collectively, our study revealed the unappreciated dual role of METTL3 as an m^6^A writer and a transcription regulator, which worked together in the same signaling pathway to drive colorectal cancer malignancy.

## Introduction

Among over 150 known RNA modifications, N6-methyladenosine (m^6^A) is the most prevalent internal decoration of mammalian messenger RNA, accounting for more than 80% of RNA modifications [1]. Since fat mass and obesity-associated protein (FTO) were identified with the ability of demethylase [2], the process of m^6^A modification has been characterized by dynamics and reversibility. Methyltransferase-like 3 (METTL3), METTL14, WT1-associated protein (WTAP) and other accessory proteins constitute heterocomplex to function as m^6^A methyltransferases (writers) in mammalian cells [3]. Among them, METTL3 serves as the core catalytic subunit of the methyltransferase complex [4]. Moreover, AlkB Homolog 5 (ALKBH5) and FTO are reported as RNA demethylases that act as erasers for recognizing different m^6^A sites [5]. In addition to writers and erasers, the fate of the RNA transcripts harboring m^6^A modifications is usually determined by the reader proteins that are in charge of decoding m^6^A markers. The list of m^6^A reader proteins includes the YTH domain family proteins (YTHDF1/2/3, YTHDC1/2) [6], the insulin-like growth factor 2 mRNA-binding proteins (IGF2BP1/2/3) [7], and the heterogeneous nuclear ribonucleoproteins (hnRNPA2B1, hnRNPG) [8, 9], etc. It is becoming clear that almost all aspects of RNA metabolism, from transcription, splicing, export, and translation to degradation, are regulated by different m^6^A reader machinery [10].

Benefiting from the development of anti-m^6^A immunoprecipitation and high-throughput sequencing, the understanding of biological functions of m^6^A modification in mRNA has been widely expanded in recent years. It has been reported that m^6^A modification is implicated in a series of biological processes, including stem cell self-renewal and differentiation [11], DNA damage repair [12], immunoregulation [13], sex determination [14], etc. Except for normal physiological activity, m^6^A has also been linked to the pathogenesis of several diseases, including cancer. In leukemia, FTO inhibition sensitizes leukemia cells to T-cell cytotoxicity and overcomes immune evasion [15]. ALKBH5 is required for the development and maintenance of leukemia stem cells by post-transcriptional regulation of several oncogenes [16]. Recently, METTL3 was reported to play an oncogenic role in various cancers, providing an opportunity for the development of effective targeted therapeutics. Knockdown of METTL3 in hepatocellular carcinoma substantially abolished cancer cell tumorigenicity and lung metastasis through augmenting SOCS2 expression relied on the YTHDF2-dependent pathway [17]. METTL3 upregulation in gastric cancer stimulated m^6^A modification of HDGF mRNA and enhanced HDGF mRNA stability, resulting in elevated tumor angiogenesis and glycolysis [18]. In colorectal cancer, higher METTL3 expression in metastatic tissues was responsible for elevated SOX2 expression, which contributed to colorectal cancer cell stemness and malignancy [19]. However, intestinal bacteria, *Fusobacterium nucleatum* induced colorectal cancer progression by activating YAP-mediated METTL3 downregulation [20]. Therefore, the potential roles of m^6^A modification and sophisticated mechanisms underlying colorectal cancer progression should be further elucidated.

This study revealed the upregulation of METTL3 expression and m^6^A level in colorectal cancer compared with normal tissues. Methylated RNA (m^6^A) sequencing showed that METTL3 depletion directly abolished JAK1 m^6^A deposition, and impaired YTHDF1-mediated JAK1 translation. Furthermore, chromatin immunoprecipitation demonstrated that METTL3 redistributed to STAT3 promoter and synergized with NF-κB to facilitate STAT3 transcription. The combined effects finally contributed to p-STAT3 signaling pathway activation and colorectal cancer cell proliferation and invasion.

## Materials and methods

### Cell culture

LoVo colon cancer cells and 293T cells were kindly provided by Shanghai Cell Collection, Chinese Academy of Sciences. The HCT116 colon cancer cell line was a gift from Zhang’s laboratory, at Zhejiang University. HCT116 and 293T cells were cultured in Dulbecco’s modified Eagle’s medium (Gibco, USA), and LoVo cells were cultured in RPMI 1640 medium (Gibco, USA) supplemented with 10% fetal bovine serum (Gibco, USA) at 37 °C in a humidified atmosphere with 5% CO_2_.

### Cell proliferation and colony formation assays

About 2000 cells were seeded into each well of a 96-well plate. At different times after cell seeding, each well was replaced with fresh medium containing 10% MTS substrate (Promega, USA), followed by incubation at 37 °C for 2 h. Then, the light absorbance value of the plate at 490 nm was recorded by a microplate reader, and the cell proliferation rate was calculated.

For cell colony formation assays, 2000 cells were seeded into each well of a six-well plate. After one week, the cells were fixed with 4% paraform and stained by crystal violet solution at room temperature for 20 min. The number of cell colonies was documented under a microscope.

### Transwell

In an 8 μm pore size transwell system (Corning, USA), the upper chamber was seeded with 5 × 10^4^ cells cultured in 500 μl serum-free medium, and the bottom well was added with 1 ml 10% complete culture medium. About 48 h after incubation in an incubator, the cells were fixed with 4% paraform and stained with crystal violet solution. A cotton swab was used to remove the cells that had unfinished translation and the rest of the cells were pictured under the microscope.

### Cell cycle investigation

About 1 × 10^6^ cells were fixed in pre-cooled 70% ethanol solution and stored under –20 °C overnight. After rinse with PBS, cells underwent Rnase A (20 µg/ml) digestion for 40 min at 37 °C, followed by incubation with propidium iodide (Sangon, Shanghai, China) at 37 °C for 30 min.

### Western blot

Cells were lysed with Western/IP lysis buffer (Beyotime, Beijing, China) and subjected to sonication. After centrifugation, the protein lysate was separated in 10% sodium dodecyl-sulfate polyacrylamide gel electrophoresis and electrotransferred to a 0.4 μm polyvinylidene fluoride membrane (Millipore, USA). About 2 h after blocking with 5% skim milk solution, the membrane was incubated with primary antibodies at 4 °C overnight. Subsequently, the membranes were incubated with secondary antibodies modified by horse radish peroxidase (Yeasen, China) for 2 h at room temperature. The amount of target protein was detected using an ECL chromogenic solution (Yeasen Biotech Corporation, Shanghai, China).

### Quantitative real-time PCR

Cellular total RNAs were extracted using an RNA isolation kit (Yeasen, China), and the complementary DNAs were synthesized with a Reverse Transcription kit (Yeasen, China) according to the manufacturer’s instructions. The expression level for each gene was detected using SYBR Master Mixture with specific primers (sequences were listed in Supplemental Table 1). The relative expression levels of genes were calculated using the ΔΔCt method and normalized with GAPDH (Glyceraldehyde-3-phosphate dehydrogenase).

### Stable knockdown and knockout cell line establishment

For RNA interference, the plasmids of psPAX, pMD2G, and shRNA-containing constructs (Vigene Biosciences, USA) were co-transfected into 293T cells. At 48 h after transfection, the cell culture supernatant was collected and filtered with 0.22 μm membrane. After concentration with a 100kD millipore filter, the virus liquid was added to the cancer cell culture medium, allowing virus infection for 24 h. The cells that successfully expressed shRNAs were screened out using puromycin treatment (MCE, USA) at the concentration of 5 μg/ml. The sequences for shRNAs were provided in supplemental Table 2.

The METTL3 knockout cell line was established using the CRISPR-Cas9 system. Briefly, the plasmids of psPAX, pMD2G, and lenti-CRISPRv2 containing guide RNA sequence (5’-CTCTGATCTGGCCTTAACAT-3’) (Vigene Biosciences, USA) were co-transfected into 293T cells. The next steps were totally the same as those above-mentioned lentivirus-mediated shRNA transfection. After puromycin treatment, the cells were seeded into a 10 cm^2^ cell culture dish and allowed the cells to establish individual colonies. Then, each cell colony was picked up and seeded into a new 96-well plate. Finally, a western blot was used to detect the knockout efficiency.

### m^6^A dot blot

After RNA isolation, the concentrations of RNA samples were quantified using Nanodrop 2000 (Thermo Fisher, USA) and an equal amount of RNA was loaded onto a nylon membrane (GE Healthcare, U.K). The membrane was cross-linked under 3 J/cm^2^ UV and blocked in RNase-free PBST (0.1% Tween-20 in 1x PBS) containing 5% non-fat milk for 1 h. Rabbit anti-m^6^A antibody (Beyotime Biotechnology, Beijing, China) diluted 1:1000 in PBST was added to the membrane and incubated overnight at 4 °C. After rinsing with PBST, the membrane was incubated with secondary anti-rabbit antibodies diluted 1:5000 in blocking buffer for 2 h at room temperature. The amount of RNA was imaged using an ECL chromogenic solution (Yeasen, China).

### m^6^A RNA immunoprecipitation

m^6^A RNA immunoprecipitation was conducted according to others’ methods described earlier (Visvanathan et al., 2017). Briefly, anti-m^6^A antibodies were coupled with Protein A/G agarose beads (Beyotime, Beijing, China) in 200 µl 1 M IP Buffer (10 mM sodium phosphate,1 M NaCl, 0.05% Triton-X) for 4 h at 4 °C. The RNA samples were denatured by heating at 75 °C for 5 min and cooled on ice for 2 min immediately. After rinse with 140 mM IP Buffer (10 mM sodium phosphate, 140 mM NaCl, 0.05% Triton-X) 3 times, the antibody-coupled beads were mixed with RNA samples and incubated together overnight at 4 °C. After incubation, the beads were washed with 140 mM IP Buffer for two times and subjected to RNA isolation using a kit (Yeasen, Shanghai, China).

### RNA associated immunoprecipitation

After centrifugation, about 1 × 10^7^ cells were collected and lysed in 500 μl RNase-free RIPA lysis buffer (Bioyetime, China) containing 1U/ul RNase inhibitor (Promega, USA) on a rotator at 4 °C for 4 h. After lysis, cell lysate was collected by centrifuging at 15,000 × *g* for 10 min and divided into two aliquots. One aliquot was incubated with YTHDF1 antibodies (Beyotime, China) overnight at 4 °C, and another was incubated with equal IgG isoforms as a control. After incubation, the RNA-protein complex was captured by Protein A/G agarose beads (Beyotime, China) by incubation at 4 °C for 3 h. After washing with RIPA lysis buffer five times, the RNA fraction was purified from the sedimented agarose beads using a Trizol-RNA isolation kit (YEASEN, China), followed by quantitative real-time PCR.

### ChIP assay

ChIP assays were performed using a ChIP assay kit (Beyotime, China) according to the manufacturer’s instructions. Briefly, about 5 × 10^6^ cells were cross-linked using 1% formaldehyde for 10 min at 37 °C. The cell lysate was subjected to sonication for chromatin fragmentation. After sonication, the DNA-protein complex was immunoprecipitated by METTL3 antibodies (Abmart Medical Technology Co., Shanghai, China), and the DNA fraction was purified using a DNA isolation kit (Beyotime, China). The primer sequences used for PCR were listed in the supplemental information.

### RNA degradation assays

To determine the RNA degradation ratio of JAK1 mRNA, Actinomycin D was added to the cell culture medium at the concentration of 3 μg/ml to block cellular de novo synthesis of RNA. The cell samples were collected at indicated time points and investigated by quantitative real-time PCR.

### Protein stability

To exclude the influence of protease-mediated protein degradation, MG132 (final concentration 50 μg/ml, MCE, USA) was added into the cell culture medium to inhibit protease activity for 8 h. Then, the expression levels of the target protein were detected by western blot. The loading amount for each sample was normalized to actin.

### Luciferase assays

The STAT3 promoter sequence containing upstream 450 bp from the transcription start point and the first exon was cloned into pGL3 plasmid. Simultaneously, the same plasmid with a mutation located in NF-κB binding site was also constructed. Then, the wild-type or the mutant construct with pRL-TK plasmid was co-transfected into HCT116 cells with 70% confluence. After 48 h, the luciferase activity was investigated using a Dual-Luciferase Reporter Gene Assay Kit (Yeasen, Shanghai, China) according to the manufacturer’s instructions.

About 500 bp JAK1 mRNA 3’UTR sequence enriched potential m^6^A sites were cloned into pmir-GLO vector (Promegra, USA). Each mutant plasmid with an A-G base substitution occurred in the m^6^A motif and was also constructed, respectively. After cell confluence up to about 70%, the pmir-GLO vectors were transfected into HCT116 cells with or without METTL3 or YTHDF1 deficiency. The luciferase activity was detected following the above-mentioned STAT3 promoter detection method.

### Animal models

About 1 × 10^7^ cancer cells suspended in 800 μl PBS were mixed with matrigel (Yeasen, China) at the volume ratio of 4:1. Then, the cell mixture was administered into 4-week-old female BALB/c nude mice by subcutaneous injection (*n* = 5). The width and length of tumor burdens were monitored weekly. When the tumor length was larger than 1 cm, all of the mice were sacrificed and the solid tumors were collected for the next investigation.

In order to establish a cell pulmonary metastasis mouse model, about 5 × 10^6^ cancer cells were diluted into 1000 μl physiological saline solution, and 200 μl cells were injected into 4-week-old female BALB/c nude mice through tail vein (*n* = 3). After about 5 weeks to allow cancer cell proliferation, mice were killed and their lungs were subjected to immunohistochemical staining. The metastasis foci presented in the lung were counted under a microscope.

### Online data mining

The expression levels and co-relationship analysis of METTL3, YTHDF1, JAK1, STAT3, CCND1, and VEGFA in clinical specimens were accomplished through interrogating GEPIA online database (http://gepia.cancer-pku.cn/) [21], TNM plotter database (https://www.tnmplot.com/) [22] and TIMER database (http://timer.cistrome.org/) [23].

### Statistic analysis

The software GraphPad Prism 8 was used for data processing and analysis. Sample with two groups was analyzed with Student’s *t*-test and samples with more than two groups were calculated with One-way ANOVA. When *p* < 0.05, the results were regarded as statistically significant.

## Results

### The upregulation of m^6^A and METTL3 in colorectal cancer

**Fig. 1:**
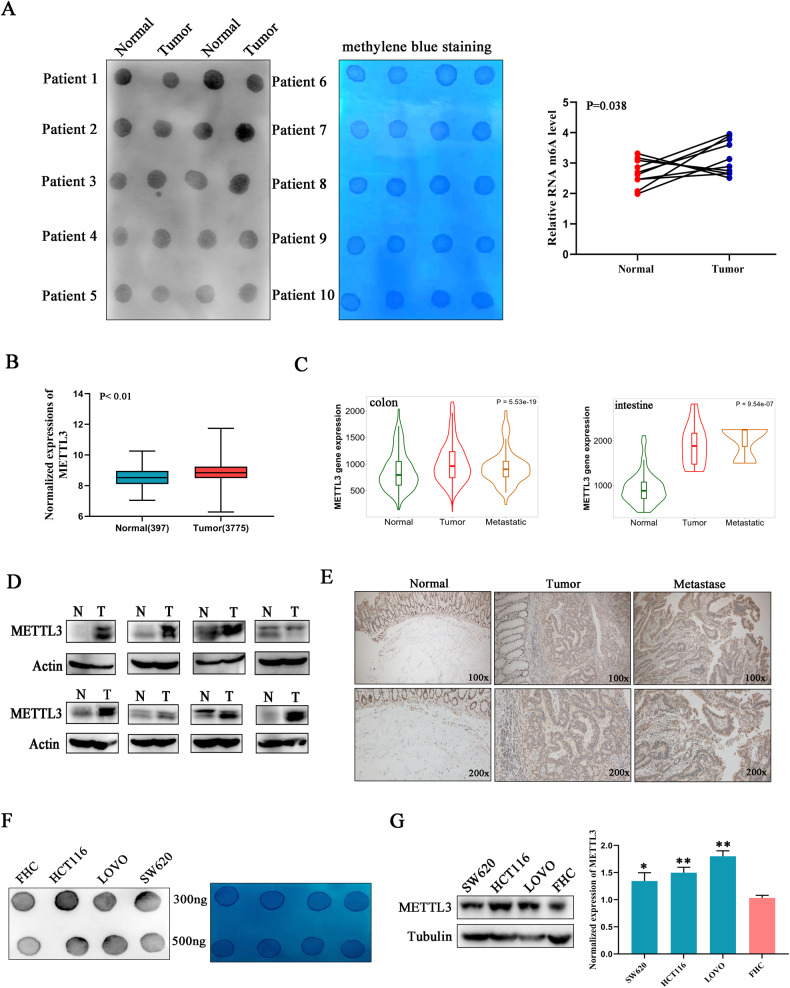
The upregulation of m^6^A and METTL3 in colorectal cancer. **A** The RNA m^6^A levels in colorectal cancerous tissues from patients were investigated using dot blot. Image J was used to analyze the amount of RNA modified with m^6^A. **B** METTL3 mRNA expression levels in normal and colorectal cancerous tissues were analyzed with the help of an online database (http://gent2.appex.kr/gent2/). **C** Relative expression alternations of METTL3 in cancerous and metastatic tissues compared with the matched normal tissues were plotted (http://kmplot.com/analysis/). **D**, **E** The expression of METTL3 on protein level was investigated in clinical samples derived from patients with colorectal cancer. **F** The m^6^A level in total RNA purified from different cell lines was detected using a dot blot. The quantity for each loaded RNA sample was normalized by methylene blue staining (right). **G** METTL3 expression was upregulated in colorectal cancer cell lines compared with normal cells (FHC).

In order to explore the role of m^6^A machinery in colorectal cancer, the total RNA m^6^A levels in 10 paired cancer and normal tissues sectioned from patients were detected using Northern dot blot. Software analysis showed that m^6^A level was upregulated in cancer tissues compared with normal tissues, suggesting the onco-promoting role of m^6^A in colorectal cancer progression (Fig. 1A). Subsequently, bioinformatics-based screening was employed to investigate the expressions of methyltransferases and demethylases (METTL3/14, WTAP, FTO, and ALKBH5) in human colorectal cancer, and we found only METTL3, the catalytic core of methyltransferases, showed significant upregulation in cancerous tissues compared with normal tissues (Fig. 1B and Fig S1A). What’s more, metastatic lesions showed higher METTL3 expression than that in primary tumors (Fig. 1C), highlighting the co-relationship between METTL3 and malignancy. Then, the expression of METTL3 in clinical samples at the protein level was evaluated through western blot and immunohistochemical staining (Fig. 1D, E). In accordance with the mRNA analysis conclusion, an obvious increase of METTL3 at the protein level was observed in colorectal cancerous and metastatic samples compared with the paired normal tissues. Besides, compared with normal human colon epithelial cells (FHC), all of the detected human colorectal cancer cell lines showed increased METTL3 expression and upregulated m^6^A modification levels (Fig. 1F, G). All the above-mentioned data indicated that METTL3 and m^6^A upregulation is associated with colorectal cancer malignancy and progression.

### The onco-promoting role of METTL3 in colorectal cancer cells

**Fig. 2:**
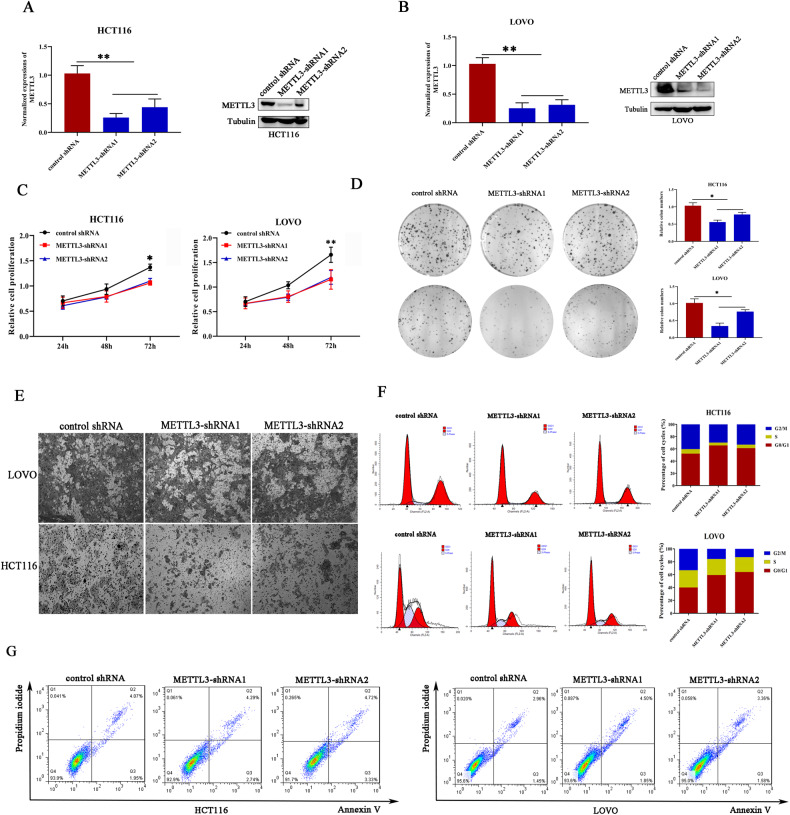
The onco-promoting role of METTL3 in colorectal cancer cells. **A**, **B** METTL3 stably silenced cell lines were established in HCT116 and LOVO cells. **C** The effect of METTL3 knockdown on cancer cell proliferation was investigated using MTT assays. “*”*p* < 0.05; “**”*p* < 0.01. **D** The colon formation ability of cancer cells was diminished in cancer cells with deleted METTL3. **E** Transwell assays were employed to evaluate the migration ability of cancer cells with or without METTL3 knockdown. **F** METTL3 silence induced cell cycle arrest compared with control cells. **G** The influence of METTL3 knockdown on cell apoptosis detected by flow cytometry.

To further elucidate the mechanisms underlying METTL3-mediated m^6^A-targeted gene regulation, colorectal cancer cells with stable decreased METTL3 were established using lentivirus-mediated shRNA transfection. The knockdown efficiency for METTL3 was demonstrated in HCT116 and LoVo cell lines using quantitative real-time PCR and western blot (Fig. 2A, B), respectively. In order to reveal the influence of METTL3 silence on cell proliferation, colony formation, and MTT assays were performed. Obvious inhibitory effects on cell proliferation and colony formation were observed in cancer cells with decreased METTL3 compared with the control group (Fig. 2C, D). Additionally, the role of METTL3 in regulating cancer cell migration was investigated. Transwell assays revealed that METTL3 knockdown significantly impaired the migration ability of HCT116 and LoVo cells (Fig. 2E). Cell proliferation inhibition is usually caused by cell cycle arrest or cell death. Therefore, cell cycle and cell apoptosis were detected in cancer cells through flow cytometry. Compared with the control group, more cells were restrained in G1/G0 stage and the fraction of cells that stayed in the G2/M stage was significantly decreased in METTL3-silenced cells (Fig. 2F), but no obvious difference in cell apoptosis was observed between these groups (Fig. 2G). Therefore, METTL3 may perform onco-promoting effects through regulating cell cycle and migration, but not dependent on cell apoptosis control.

### The influence of METTL3 silence on JAK1/STAT3 signaling pathway

**Fig. 3:**
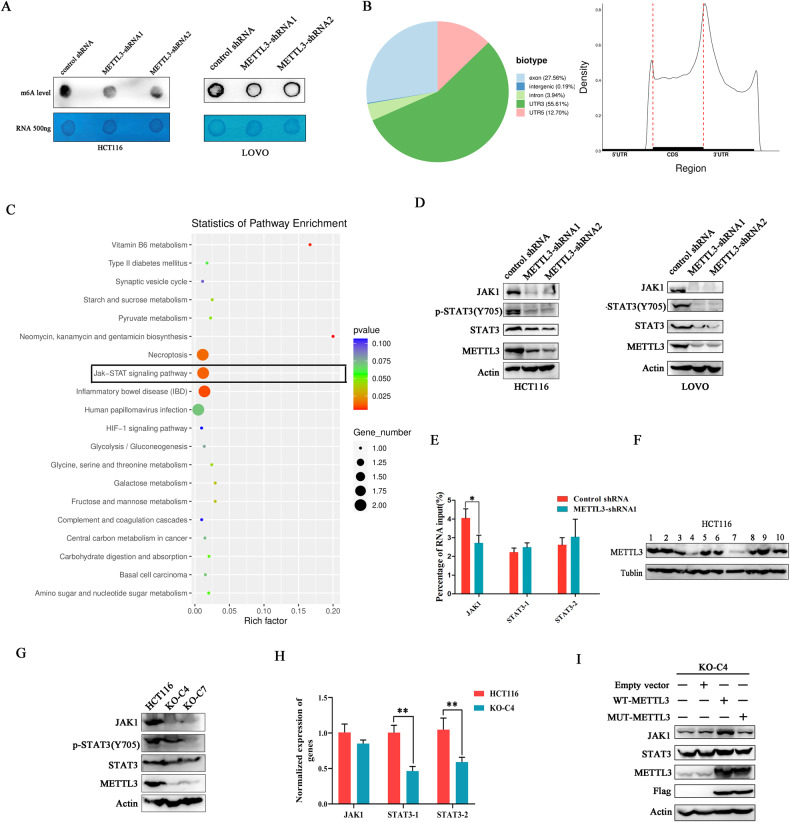
The influence of METTL3 silence on JAK1/STAT3 signaling pathway. **A** The RNA m^6^A level was decreased in METTL3-silenced cells compared with control cells. **B** The m^6^A deposition pattern on mRNAs was deciphered using MeRIP sequencing. **C** Pathway enrichment analysis was performed subjected to genes with altered m^6^A peaks. **D** METTL3 knockdown down-regulated JAK1/STAT3 axis activity. **E** The influence of METTL3 knockdown on mRNA m^6^A modification of JAK1 and STAT3. **F** METTL3 knockout cells were established using the CRISPR-cas9 method. **F** JAK1/STAT3 signaling pathway activity was inhibited in METTL3 knockout cells. **G** The influence of METTL3 knockout on the JAK1/STAT3 signaling pathway was detected by western blot. **H** The mRNA expression levels of JAK1 and STAT3 were investigated in METTL3 knockout cancer cells. Two pairs of primers targeting different STAT3 encoding sequences were designed. **I** Wild-type and methyltransferase-dead METTL3 (mutant type) were re-expressed in METTL3 knockout HCT116 cancer cells.

Considering the function of METTL3 on RNA modification, the RNA m^6^A level in cancer cells with METTL3 deficiency was investigated through dot blot. After METTL3 silencing, the m^6^A level was significantly decreased in cancer cells compared with a control group (Fig. 3A), which is in accordance with others’ previous reports. To decipher the role of METTL3 on m^6^A target gene expression, methylated RNA immunoprecipitation and RNA sequencing (MeRIP-seq) were performed in HCT116 cells with or without METTL3 depletion. The sequencing results showed that the majority of m^6^A peaks were distributed in mRNA 3’UTR and the site near the translation stop point with the highest m^6^A deposition (Fig. 3B), which is consistent with previous sequencing results. The genes with changed m^6^A peaks (log2 ≥ 1) were picked up and subjected to pathway enrichment statistics. JAK/STAT signaling pathway showed up and attracted our attention (Fig. 3C). The status of the JAK1/STAT3 signaling pathway was investigated and the western blot results demonstrated METTL3 silencing significantly inhibited STAT3 phosphorylation at Tyr705 residue. Surprisingly, an obvious decrease in JAK1 and STAT3 expressions was also observed in METTL3 knockdown cancer cells, but not in control groups (Fig. 3D). To make it clear whether METTL3 regulates JAK1 and STAT3 expression is dependent on its methyltransferase activity, the RNAs harboring m^6^A modification were sedimented using anti-m^6^A antibodies and subjected for quantitative real-time PCR. To exclude the potential of off-target, two paired primers targeted STAT3 at different sites were designed. Real-time PCR results showed the amount of JAK1 mRNA with m^6^A modifications was decreased after METTL3 knockdown, but there is no difference for m^6^A-installed STAT3 mRNA (Fig. 3E), indicating that METTL3 possibly mediated STAT3 expression in a m^6^A-independent manner.

Subsequently, CRISPR-Cas9 technology was employed to build METTL3 knockout cells in HCT116. After single cell selection, ten cell colons were investigated using western blot, and found only colon-4 (KO-C4) and colon-7 (KO-C7) showed METTL3 knockout efficiency (Fig. 3F). For JAK1/STAT3 pathway analysis, western blot detection in METTL3 knockout cells showed the same results as that observed in METTL3 knockdown cells (Fig. 3G). Real-time PCR investigation found METTL3 knockout significantly down-regulated STAT3 expression at the mRNA level but had no effect on JAK1 mRNA expression (Fig. 3H). To further verify that METTL3-mediated regulation on JAK1 and STAT3 is not the effects of off-target, we exogenously overexpressed wild-type METTL3 (WT-METTL3) and mutant METTL3 (D395A/W398A) that lost methyltransferase activity in METTL3 knockout cells. We have found that either WT-METTL3 or mutant METTL3 overexpression rescued the expression of STAT3, but only the WT-METTL3 rescued JAK1 expression, the effects of mutant METTL3 overexpression on JAK1 are compromised (Fig. 3I). All above-mentioned data indicated that METTL3 regulated JAK1 expression at protein level but not on its mRNA level in a m^6^A dependent manner and influenced STAT3 mRNA expression, which corporately promoted the activation of JAK1/STAT3 signaling pathway in colorectal cancer cells.

### YTHDF1 promoted JAK1 mRNA translation in a m^6^A-dependent manner

**Fig. 4:**
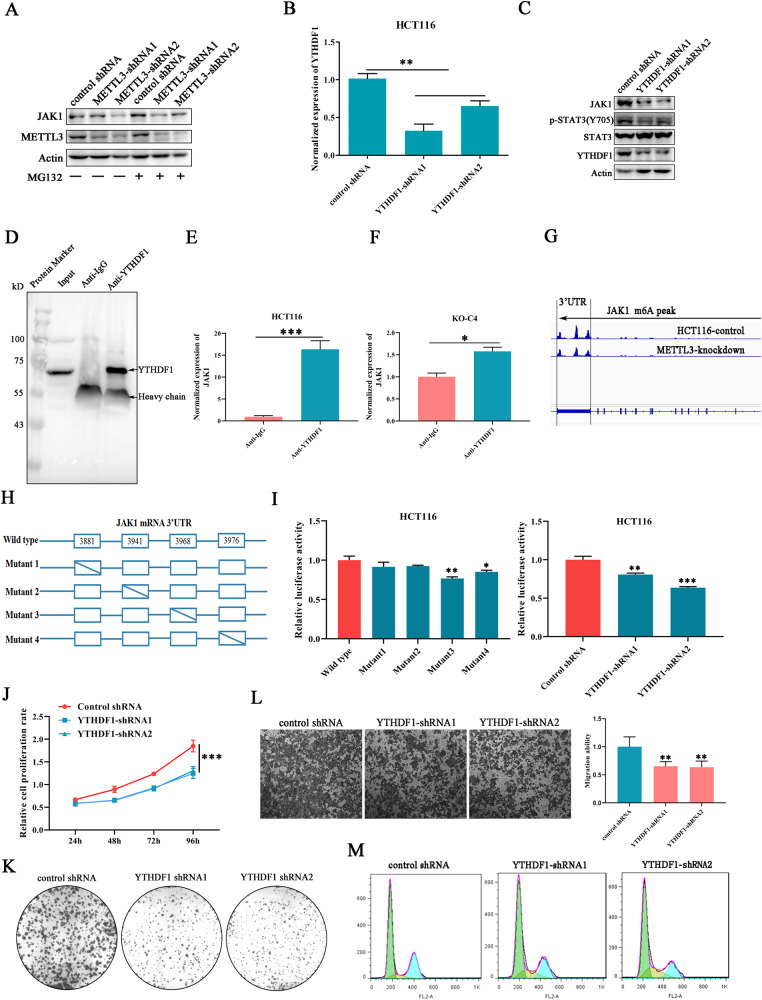
YTHDF1 promoted JAK1 mRNA translation in a m^6^A-dependent manner. **A** METTL3-regulated JAK1 expression is independent of proteasome-mediated protein degradation. MG132, a protease inhibitor. **B** YTHDF1-silenced cell lines were established using lentivirus-mediated shRNA transfection. **C** The influence of YTHDF1 knockdown on JAK1 expression. **D** YTHDF1 was specifically immunoprecipitated by antibodies anchored on agarose beads. **E**, **F** The level of JAK1 mRNA in YTHDF1 sediments was investigated in cancer cells with or without METTL3 depletion. **G** The distribution pattern of peaks with m^6^A in JAK1 mRNA was analyzed by referring to MeRIP-sequencing data. **H** Schematic illustration of different JAK1 3’UTR constructs, comprising wild-type, and mutations of the m^6^A motifs in m^6^A peaks (Mutant1, Mutant2, Mutant3, and Mutant4). **I** Dual-luciferase reporter assays for the wild type or mutant JAK1 3’UTR sequence in cancer cells with or without silenced YTHDF1. **J**, **K** The influence of YTHDF1 knockdown on HCT116 cancer cell proliferation and colony formation was detected. **L** YTHDF1 knockdown impaired cancer cell migration detected by Transwell assays. **M** The number of cells at mitotic phase was diminished in YTHDF1-silenced cancer cells.

The finding of METTL3 deficiency induced JAK1 downregulation on protein level, but not on mRNA, prompted us to address whether METTL3 participated in regulating JAK1 translation or protein stability. Therefore, MG132, an inhibitor of protease, was used to block the proteasome-mediated protein degradation. We have found that MG132 treatment failed to complement METTL3 knockdown-mediated JAK1 downregulation (Fig. 4A), indicating that METTL3 may be involved in JAK1 translation. A large amount of evidence has demonstrated that YTHDF1 was able to promote target RNA translation by recognizing their m^6^A sites [24]. Then, cells with stable silenced YTHDF1 were constructed in the HCT116 cell line (Fig. 4B). Western blot investigation revealed that YTHDF1 knockdown efficiently inhibited STAT3 phosphorylation and reduced the expression level of JAK1, but had no influence on STAT3 expression (Fig. 4C). To make it clear whether YTHDF1 regulated JAK1 expression through directly binding with JAK1 mRNA. RNA-associated immunoprecipitation (RIP) and quantitative real-time PCR were conducted (Fig. 4D). We have found that JAK1 mRNA was enriched in YTHDF1 immunoprecipitates and the interaction was obviously abrogated after METTL3 knockout (Fig. 4E, F), highlighting the indispensability of m^6^A in YTHDF1 and JAK1 mRNA interaction. Through scanning MeRIP-seq data in HCT116, numerous m^6^A peaks with very high confidence were distributed in JAK1 mRNA 3’UTR and four potential m^6^A sites were predicted (Fig. 4G). Then, the sequence of JAK1 3’UTR was cloned into a Dual-luciferase pmirGLO vector, and mutant vectors with single base substitution occurred in potential m^6^A site were also constructed, respectively (Fig. 4H). Luciferase assay results showed that the luciferase activity was significantly impaired in cells transfected with vectors harbored m^6^A mutations at site3 and site4 compared with wild-type group, implying that the m^6^A modifications located in JAK1 mRNA 3’UTR site3 and site4 are responsible for m^6^A-mediated JAK1 post-transcriptional regulation (Fig. 4I). In addition, YTHDF1 deficiency also induced an obvious fluorescence decrease compared with control group (Fig. 4I), which further verified the vital role of YTHDF1 in recognizing JAK1 m^6^A sites. All above-mentioned data demonstrated that the regulatory function of METTL3 on JAK1 expression was achieved by relying on the ability of YTHDF1 to enhance target gene translation by recognizing m^6^A sites.

Then, the role of YTHDF1 in colorectal cancer cells was investigated. We found that silencing of YTHDF1 significantly inhibited cell proliferation and colony formation (Fig. 4J, K), and the cell migration ability was also impaired in cells with YTHDF1 depletion compared with control cells (Fig. 4L). Additionally, flow cytometry analysis showed that the fraction of cells that stayed in the M stage was decreased and more cells were arrested at the S stage (Fig. 4M), which was consistent with the finding observed in METTL3-silenced cells. Collectively, all the above data suggested that METTL3 relied on the binding ability of YTHDF1 with the JAK1 m^6^A site to promote JAK1 translation, resulting in STAT3 pathway activation and cancer progression.

### METTL3 and NF-κB corporately regulated STAT3 transcription

**Fig. 5:**
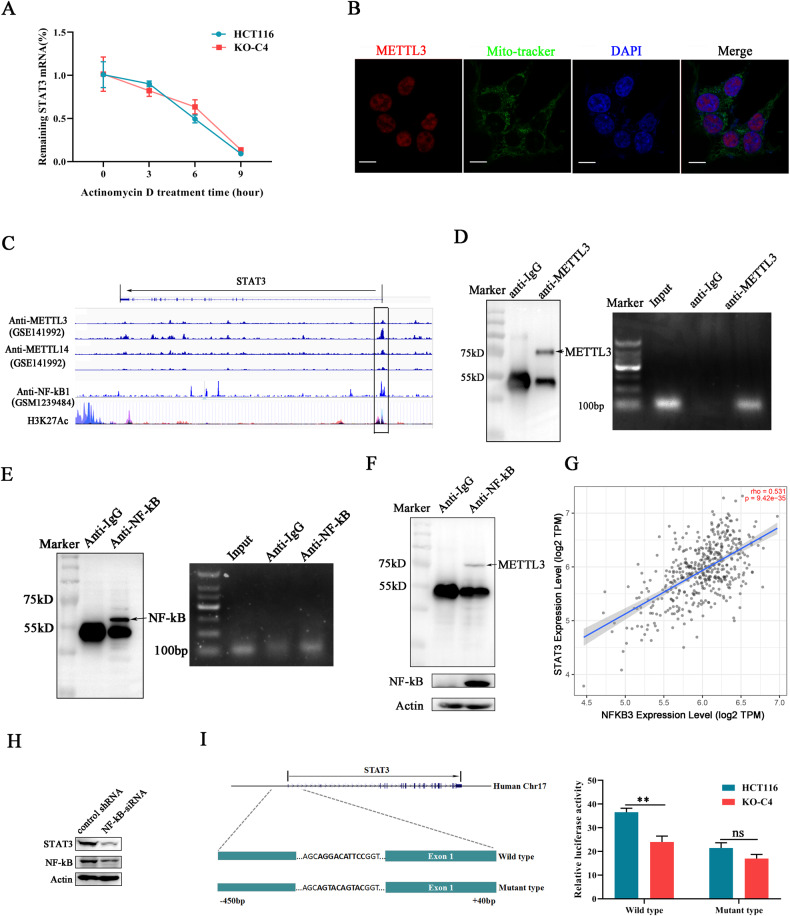
METTL3 and NF-κB corporately regulated STAT3 transcription. **A** The RNA degradation rate of STAT3 mRNA was investigated in cancer cells. Actinomycin D was used to block RNA synthesis. **B** The distribution of METTL3 in HCT116 cells was indicated under a fluorescence microscope. Mito-tracker (green) was used to mark mitochondria location. Scale bars, 10 μm. **C** The DNA peaks bound by METTL3/METTL14/NF-κB were mapped to the genome sequence encoding STAT3. **D** Chromatin Immunoprecipitation assay (ChIP) was used to investigate the interaction between METTL3 and STAT3 promoter. The efficiency of METTLE antibodies to sediment METTL3 was detected using a western blot (left), and the amount of STAT3 promoter in METTL3 sediments was investigated using PCR (right). **E** The binding ability of NF-κB with STAT3 promoter was detected by ChIP assay. **F** The interaction between METTL3 and NF-κB was detected using Co-Immunoprecipitation assays (Co-IP). **G** The co-relationship between STAT3 expression and NF-κB expression in colorectal cancer samples was analyzed through the screening TCGA database (http://timer.cistrome.org/). **H** NF-κB knockdown induced STAT3 downregulation in HCT116 cancer cells. **I** The STAT3 promoter activity was detected using dual-luciferase reporter assays. Schematic illustration of constructs containing wild-type or mutant STAT3 promoter sequence (left).

Our previous work has revealed that METTL3 deficiency induced decreased STAT3 expression at both protein and mRNA levels but had no effect on its RNA m^6^A modification. We have to speculate whether JAK1 transcription or RNA degradation was influenced by METTL3. Here, we employed Actinomycin D to interrupt cellular de novo RNA synthesis. Real-time PCR detection demonstrated that there was no obvious difference in STAT3 mRNA degradation rate between METTL3 knockout and control group under Actinomycin D exposure (Fig. 5A), which indicated that STAT3 mRNA degradation was not affected by METTL3. Recent studies reported the function of METTL3 was associated with their cellular distribution pattern [25]. Cytoplasmic location was usually associated with non-m^6^A target RNA translation control, but when METTL3 localized in the nucleus, METTL3 was not only responsible for RNA m^6^A modification but also for transcription regulation [26]. Therefore, the location of METTL3 in HCT116 cells was detected using immunofluorescence, and found that the majority of METTL3 was localized in the nucleus, barely any signal was observed in cytosol (Fig. 5B). Then, we performed bioinformatic analysis by referring the published ChIP-seq data (GSE141992) for METTL3 and METTL14, and found METTL3 binding sites are abundant in STAT3 promoter specifically near the vicinity of transcription start point (Fig. 5C). It has been reported METTL3 binding site in chromatin usually overlapped with NF-κB, and they formed heterocomplex to trans*-*regulate downstream gene transcription [26]. Surprisingly, the footprint of NF-κB was also found in the STAT3 promoter region (Fig. 5C), which indicated METTL3 may synergize with NF-κB to regulate STAT3 transcription. Subsequently, ChIP assays for METTL3 and NF-κB were performed in HCT116 cells, respectively. Paired primers specifically target the sequence near the STAT3 transcription site was designed. Through separating PCR products, we have found that the STAT3 promoter fragments were presented in METTL3 and NF-κB sediments but not in the anti-IgG group (Fig. 5D, E), which demonstrated the direct interaction between METTL3/NF-κB with STAT3 promoter.

Subsequently, the direct interaction between METTL3 and NF-κB was also demonstrated through co-immunoprecipitation (Fig. 5F). To further explore whether NF-κB with the ability to regulate STAT3 expression, the expression of NF-κB was inhibited by siRNA in cancer cells and a moderate decrease on STAT3 expression was found (Fig. 5H). Besides, online data mining revealed the expressions of NF-κB and STAT3 were positively correlated in clinicopathological colorectal cancer samples (Fig. 5G), which provided new evidence for the role of NF-κB in regulating STAT3 expression. Finally, the sequence containing the upstream 450 bp and downstream 40 bp from the transcription start site was constructed into promoter activity detection plasmid (Fig. 5I). What’s more, the same sequence with a mutation in known NF-κB binding motif was also constructed and transfected into cancer cells for promoter activity investigation. Compared with wild-type group, the NF-κB mutation significantly diminished STAT3 promoter activity, and this inhibitory effect was further extended in METTL3 knockout cancer cells (Fig. 5I). Collectively, the above data demonstrated that METTL3 and NF-κB synergistically promoted the transcription of STAT3.

### The downstream effects of p-STAT3 signaling dysregulation

**Fig. 6:**
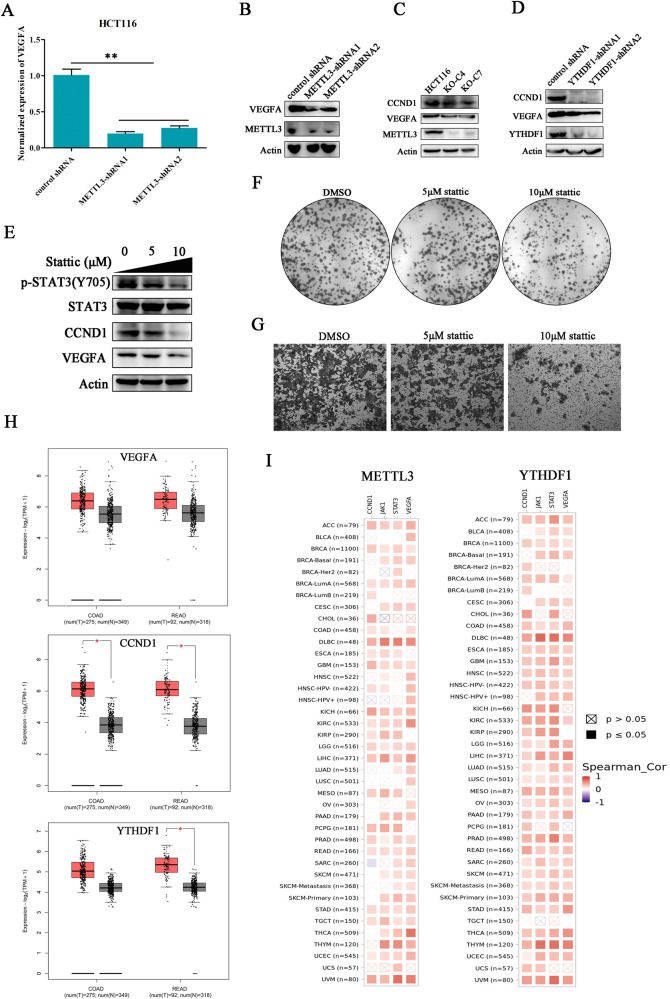
The downstream effects of JAK1/STAT3 signaling dysregulation. **A**, **B** The effects of METTL3 knockdown on VEGFA expression were investigated by quantitative real-time PCR and western blot. “**”*p* < 0.01. **C** The influence of METTL3 knockout on CCND1 expression. **D** The expression level of VEGFA and CCND1 in YTHDF1-silenced cells was investigated. **E** The inhibitory efficiency of STAT3 inhibitor (Stattic) at different concentrations was investigated in HCT116 cancer cells by western blot. **F**, **G** The influence of Stattic on HCT116 cancer cell colon formation and migration was detected. **H** The expression levels of VEGFA, CCND1, and YTHDF1 in colorectal cancer and paired normal tissues were evaluated through screening GEPIA database (http://gepia2.cancer-pku.cn/). **I** The co-relationships between METTL3 and YTHDF1 with JAK1/STAT3 signaling target genes were analyzed through referring TIMER database (http://timer.cistrome.org/).

The molecular mechanism utilized by METTL3 to regulate JAK1 and STAT3 expressions has been elucidated in colorectal cancer cells, but the downstream effector that orchestrated JAK/STAT3 signaling function in cancer cells is still obscure. It has been reported that STAT3 is able to promote angiogenesis by directly enhancing VEGFA transcription [27]. Therefore, the expression of VEGFA in METTL3-silenced cancer cells was investigated. The deficiency of METTL3 significantly inhibited VEGFA expression at both mRNA level and protein level (Fig. 6A, B). According to our findings, METTL3 silence inhibited cell proliferation by inducing cell cycle arrest. Exactly, CCDN1, a cell cycle regulator that is required for G1/S transition, was reported as a STAT3 target in several cancer types [28]. Here, we have found the expression of CCND1 was obviously decreased in METTL3-deleted cells, and the same result was also demonstrated in YTHDF1-silenced cancer cells (Fig. 6C, D). This data suggested METTL3 regulates VEGFA and CCND1 expression potentially through activating JAK1/STAT3 signaling pathway. Whether the dysregulation of the JAK1/STAT3 pathway can directly shape the characteristics of colorectal cancer cells? Stattic, a small molecular inhibitor of STAT3, was used to block the phosphorylation of STAT3 in HCT116 cancer cells. Western blot demonstrated the activity of STAT3 was inhibited by stattic in a dose-dependent manner, and the downstream factors, including VEGFA and CCND1, were also down-regulated after stattic treatment (Fig. 6E). Besides, stattic dose-dependently impaired HCT116 cancer cell colony formation and migration (Fig. 6F, G). Database analysis revealed the expressions of VEGFA, CCND1, and YTHDF1 were all upregulated in colorectal cancer tissues compared with the normal cohort (Fig. 6H). The expression cor-relationships between METTL3 and its targets, including JAK1, VEGFA, CCND1, and STAT3, was positive in almost all analyzed cancer types (Fig. 6I). In conclusion, the data above-mentioned revealed that the STAT3 signaling pathway played an important role in METTL3-mediated colorectal cancer cell proliferation and migration.

### METTL3 and YTHDF1 promoted tumor growth and metastasis in vivo

**Fig. 7:**
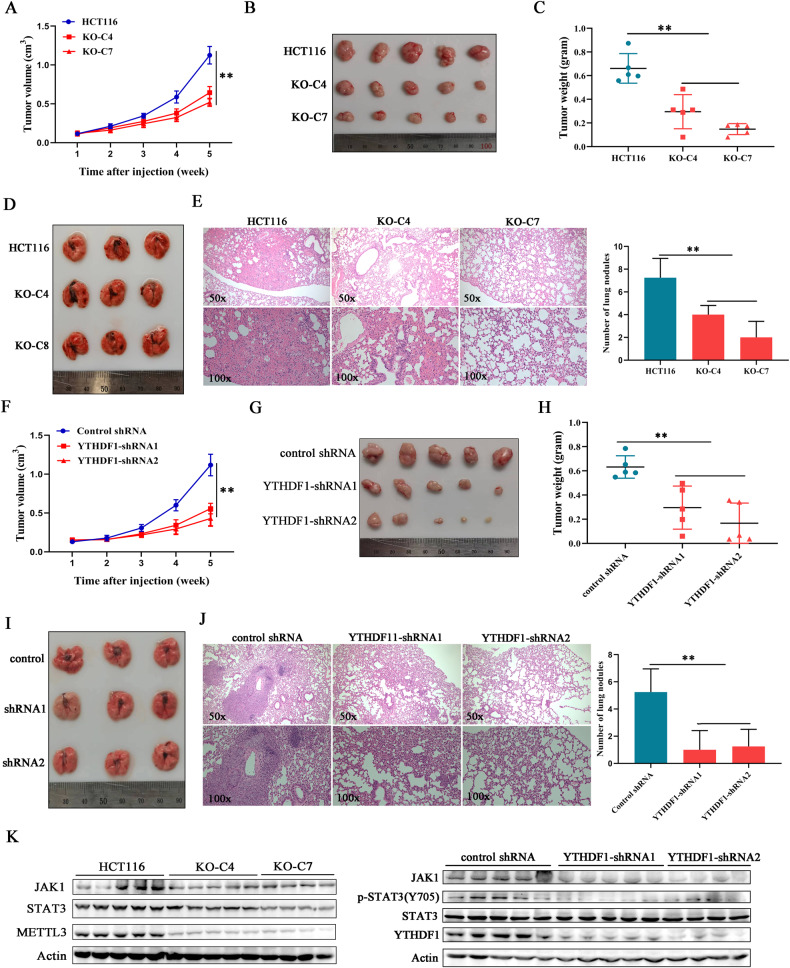
METTL3 and YTHDF1 promoted tumor growth and metastasis in vivo. **A** Time course of colorectal cancer xenograft growth. **B** Representative xenograft tumors after subcutaneous injection of HCT116 cells with or without deleted METTL3. **C** Tumor weight statistics of xenografts. **D** Representative picture of lungs sectioned from mouse metastasis model. This model was established by tail intravenous injection of METTL3 knockout HCT116 cells. **E** Mouse lung tissues were subjected to H&E staining. The number of metastases nude was calculated under a microscope. “**”*p* < 0.01. **F** Tumor growth curve was plotted at different times after YTHDF1-silenced cancer cells injection subcutaneously. **G** Representative tumors xenografted from nude mice. **H** Tumor weight statistics of xenografts. **I** Representative picture of lungs sectioned from a mouse metastasis model. YTHDF1-silenced cells were injected into nude mice through the tail vein. **J** The metastatic nudes in the lung were stained by H&E. “**”*p* < 0.01. **K** The expressions of JAK1 and STAT3 in xenografts were investigated by western blot.

In order to explore the role of METTL3 in regulating tumorigenesis in vivo, colorectal cancer cells with or without METTL3 deficiency were subcutaneously injected into BALB/c nude mice. We found that METTL3 knockout efficiently delayed tumor growth (Fig. 7A), as the weights and sizes of METTL3-deficient xenografts displayed a significant decrease compared with the compared group (Fig. 7B, C). We then explored whether METTL3 could contribute to colorectal cancer cell metastasis in vivo by establishing a pulmonary metastasis mouse model. Tail vein injection of HCT116 control cells resulted in serious pulmonary metastases, whereas deletion of METTL3 almost completely abolished metastatic node formation (Fig. 7D, E). All of this confirmed the oncogenic role of METTL3 in colorectal carcinogenesis by promoting cell proliferation and metastasis.

**Fig. 8:**
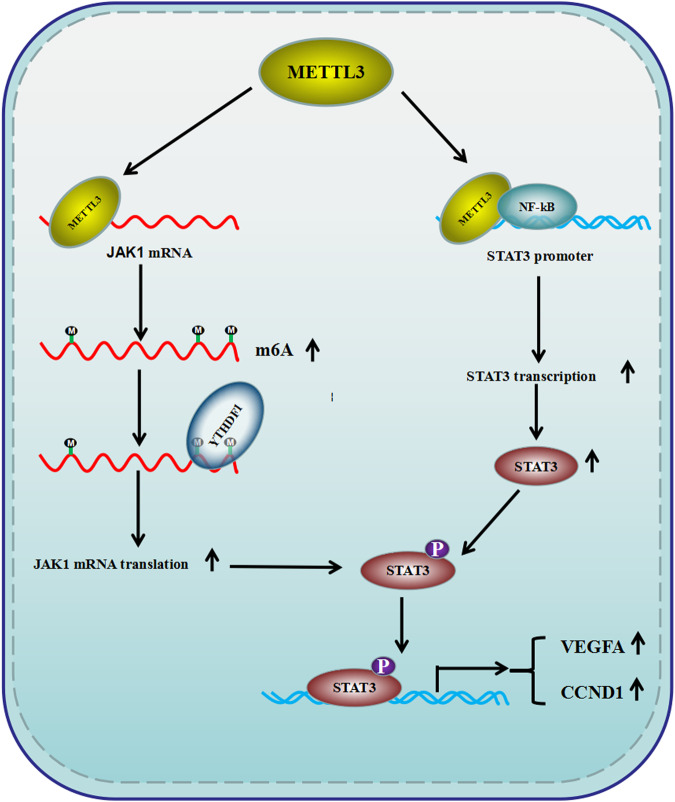
Schematic diagram for METTL3-mediated STAT3 signaling pathway activation in colorectal cancer cells. METTL3 upregulated JAK1 expression through a m^6^A/YTHDF1 axis and synergized with NF-κB to enhance STAT3 transcription, resulting in STAT3 signaling pathway activation and colorectal cancer progression.

To further confirm the conclusions obtained from ex vivo studies, cancer cells with YTHDF1 depletion were injected into nude mice to establish tumor-bearing and pulmonary metastasis mouse models. After YTHDF1 silencing, tumor burden was obviously alleviated (Fig. 7F–H), and fewer metastatic nodes were presented in lung tissues sectioned from mice injected with YTHDF1 deficient cells compared with the control group. (Fig. 7I, J). What’s more, the expression levels of STAT3 signaling-associated proteins were diminished in xenografts with METTL3 or YTHDF1 deficiency (Fig. 7K), which is consistent with the results observed in cells. Collectively, our results revealed that METTL3 simultaneously upregulated JAK1 and STAT3 expression in m^6^A-dependent and -independent manners, which contributed to STAT3 signaling activation, resulting in colorectal cancer proliferation and progression in vitro and in vivo (Fig. 8).

## Discussion

Recent studies have expanded our understanding of the mechanism underlying the function of METTL3, especially the studies linking METTL3-mediated RNA modifications to post-transcriptional and epigenetic regulation of target gene expression. Here, our study has revealed that METTL3 and RNA m^6^A level was significantly upregulated in colorectal cancer tissues compared with normal tissues, which is consistent with the emerging reports that supported the onco-promoting role of METTL3 and m^6^A in various cancers [29]. The RNA N6-methyladenosine modification is widely distributed among eukaryotic transcripts and the fates of m^6^A-modified targets are usually determined by reader proteins. Although opposite effects of METTL3 were also reported in colorectal cancer, different research backgrounds and perspectives may account for this paradox.

Methylated RNA immunoprecipitation and RNA sequencing revealed that transcripts with altered m^6^A modification after METTL3 knockdown were mainly enriched in the JAK-STAT signaling pathway, and we have found that JAK1 was a direct target of METTL3, and JAK1 translation was manipulated by YTHDF1 in a m^6^A dependent manner. In addition, STAT3 transcription was found to be regulated by the nuclear redistribution of METTL3 to STAT3 promoter, which achieved is independent of m^6^A deposition, but the synergistic effect of NF-κB is indispensable during this process. Chromatin immunoprecipitation and sequencing have revealed that the majority of METTL3 binding peaks overlapped with those bound by NF-κB [26]. A NF-κB-recognized motif within the promoter of STAT3 was identified and the positive co-relationship between STAT3 and NF-κB expression in colon cancerous tissues was demonstrated. Although METTL3 or NF-κB knockdown was able to modestly downregulate the expression of STAT3, whether there are any other key unknown factors taking part in this process needs to be further elucidated. According to the results of cell phenotype assays, the JAK1-STAT3 signaling downstream effectors, including VEGFA and CCND1, were screened out and their functions were uncovered in cancer cells, but themselves were also reported as METTL3-modified targets in a recent study [30]. In fact, we can not completely exclude the potential influence of METTL3 on these gene expressions due to the complexity and background dependency of gene regulation. As for which function mediated by METTL3, m^6^A writer or transcription regulator, dominated cancer cell biological process, and whether this mechanism is universal among other cancer types indeed has exceeded our current study.

In summary, our work has demonstrated that METTL3 promoted colorectal cancer progression through upregulating JAK1 and STAT3 expression in m^6^A-dependent and -independent manners, contributing to the activation of the p-STAT3 signaling pathway. Therefore, the combination of inhibiting p-STAT3 and METTL3 simultaneously may shed new sight on colorectal cancer therapy.

### Supplementary information

supplements

checklist

Uncropped western blots
