# Supplementary material for: METTL3 promotes colorectal cancer progression through activating JAK1/STAT3 signaling pathway
Source: Cell Death Dis. 2023 Nov 25;14(11):765. doi: 10.1038/s41419-023-06287-w (PMC10673931; doi:10.1038/s41419-023-06287-w)

1. Primers used for quantitative real-time PCR:

| GENE | Sequence(5’--3’) |
| --- | --- |
| METTL3 | F: ACCTATGCTGACCATTACCAAG  R: CTGTTGGTTCAGAAGGCTCTC |
| JAK1 | F: CTTTGCCCTGTATGACGAGAAC  R: ACCTCATCCGGTAGTGGAGC |
| STAT3 | F1: GACATTCCCAAGGAGGAGGC  R1: TATTGCTGCAGGTCGTTGGT  F2: TCTGTGTGACACCAACGACC  R2: AGGTGAGGGACTCAAACTGC |
| YTHDF1 | F: TCAGGCTGGAGAATAACGA  R: GGTTGTGTGCTTGTAGGAACT |
| VEGFA | F: CACACAGGATGGCTTGAAGA  R: AGGGCAGAATCATCACGAAG |
| CCND1 | F: GATGCCAACCTCCTCAACGA  R: ACTTCTGTTCCTCGCAGACC |
| STAT3 promoter | F: AGGGGCATTTAAAGTGCCTTGA  R: AGCCAAGAGGAGACTGATAC |

1. Sequences used for RNA interference assays:

| GENE | Sequence(5’--3’) |
| --- | --- |
| Control shRNA | CATTCCGAGTTCTAACTGCT |
| METTL3-shRNA1 | GCAAGTATGTTCACTATGAAA |
| METTL3-shRNA2 | GCCAAGGAACAATCCATTGTT |
| Control shRNA | CTAAGCTCCGGTTATCAAGC |
| YTHDF1-shRNA1 | GATACAGTTCATGACAATGA |
| YTHDF1-shRNA2 | GAAACGTCCAGCCTAATTCT |
| Control siRNA | AACTCTGGCTAGTCTGGCA |
| NF-kB siRNA | GTGACAAAGTTCAGAAAGA |

1. Guide RNA sequence used for METTL3 knockout assay：

METTL3-gRNA: 5’-CTCTGATCTGGCCTTAACAT-3’

1. Antibodies:

| Name | Brand and item number |
| --- | --- |
| Phospho-STAT3(Tyr705) Rabbit Monoclonal Antibody | Beyotime, China. AF1276 |
| STAT3 Rabbit Monoclonal Antibody | Beyotime, China. AF1492 |
| Cyclin D1 Rabbit Polyclonal Antibody | Beyotime, China. AF0126 |
| VEGFA Rabbit Polyclonal Antibody | Beyotime, China. AF0312 |
| YTHDF1 Rabbit Polyclonal Antibody | Beyotime, China. AG8382 |
| HRP-conjugated Goat anti-Rabbit IgG | Beyotime, China. A0208 |
| HRP-conjugated Goat anti-Mouse IgG | Beyotime, China. A0192 |
| METTL3 Rabbit Antibody | Abmart, China. T57240S |
| JAK1 Mouse Antibody | Abmart, China. M63209S |
| β-Actin Rabbit Monoclonal Antibody | Servicebio, China. GB15003 |
| Cy5-conjugated Goat anti-Rabbit IgG | Servicebio, China. GB27303 |
| β-Tubulin Rabbit Antibody | CST, USA. #2146S |

**Supplements for Figures**

1. Fig S1A. TCGA database analysis of methyltransferases (METTL3, WTAP, METTL14) and demethylases (FTO, ALKBH5) expressions in colon cancer.


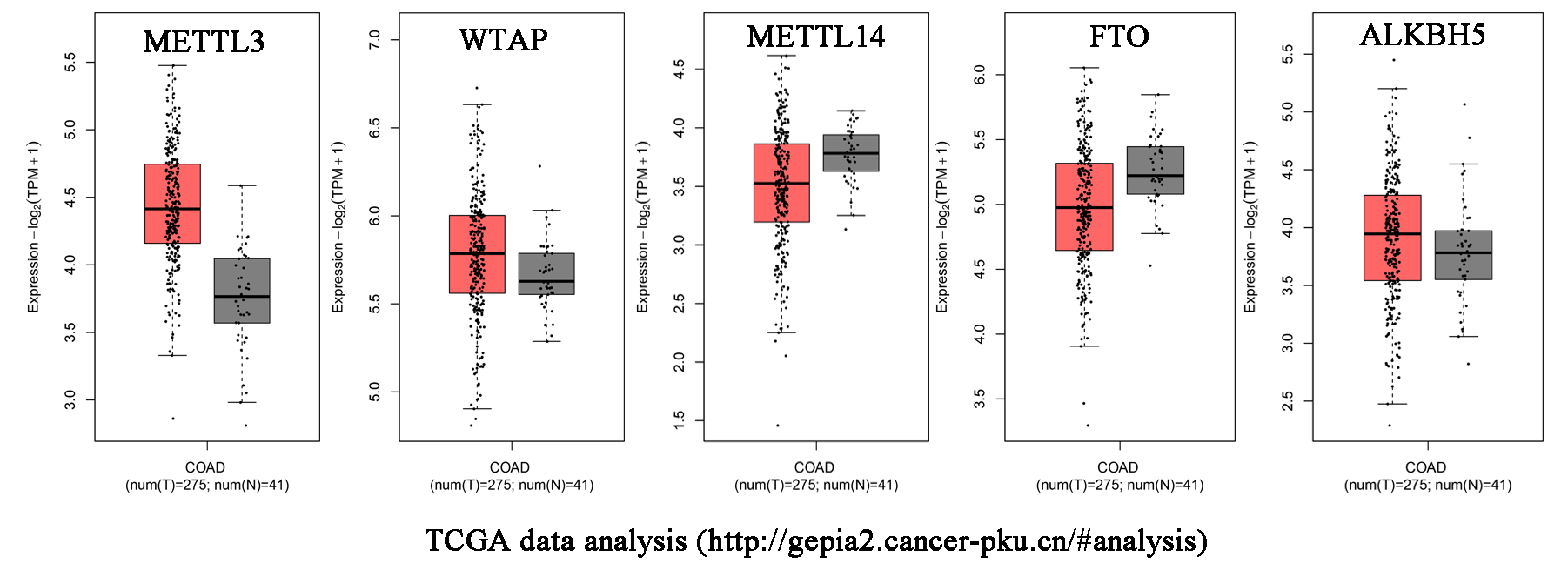


1. Fig S2A. Cell migration ability analysis for Fig 2E.


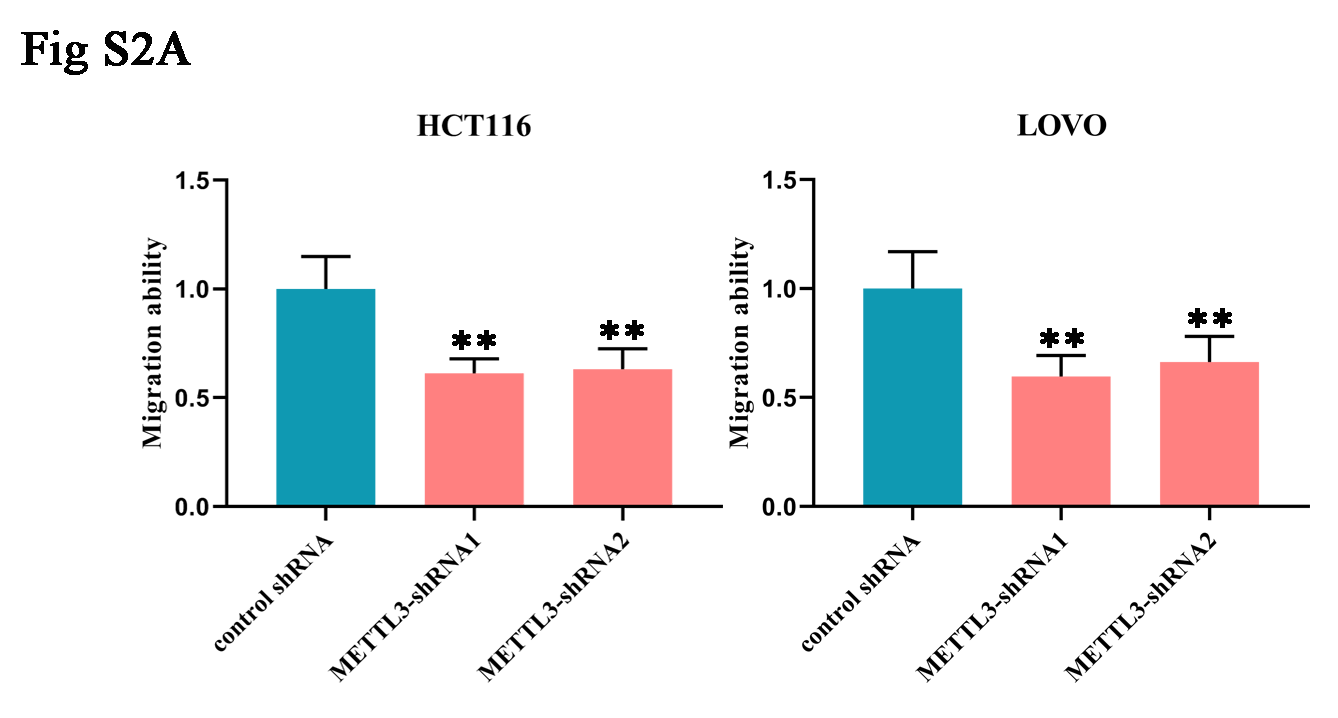


1. Fig S3A. Quantitative real-time PCR to investigate JAK1/STAT3 expression in cells with or without METTL3 depletion .


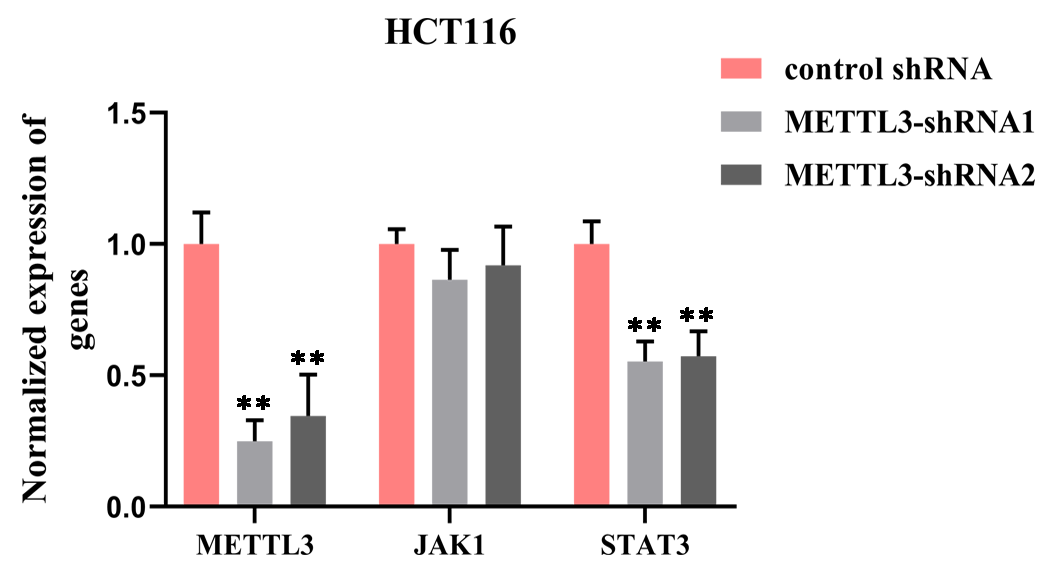


1. Fig S4K. Cell colony formation ability analysis for Fig 4K.


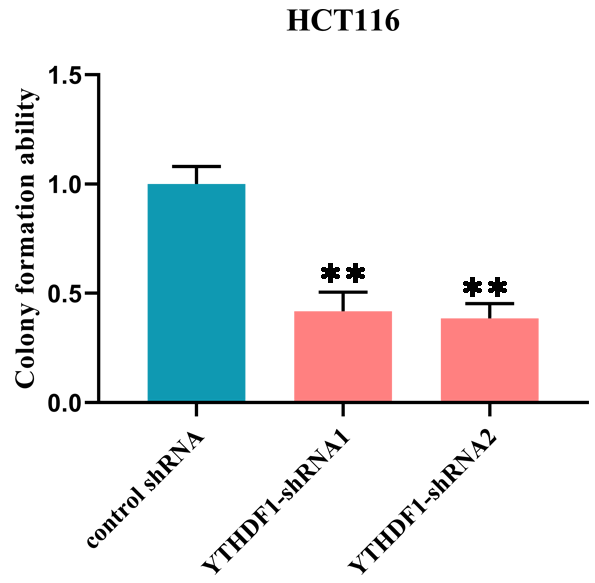

Supplement: Supplementary file 1 — supplements [file 41419_2023_6287_MOESM1_ESM.doc]
