# Supplementary material for: METTL3 promotes colorectal cancer progression through activating JAK1/STAT3 signaling pathway
Source: Cell Death Dis. 2023 Nov 25;14(11):765. doi: 10.1038/s41419-023-06287-w (PMC10673931; doi:10.1038/s41419-023-06287-w)

Fig 1A

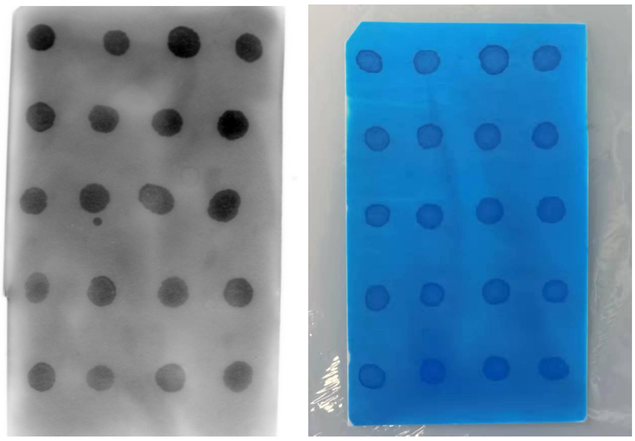

Fig 1D

Actin Mettl3

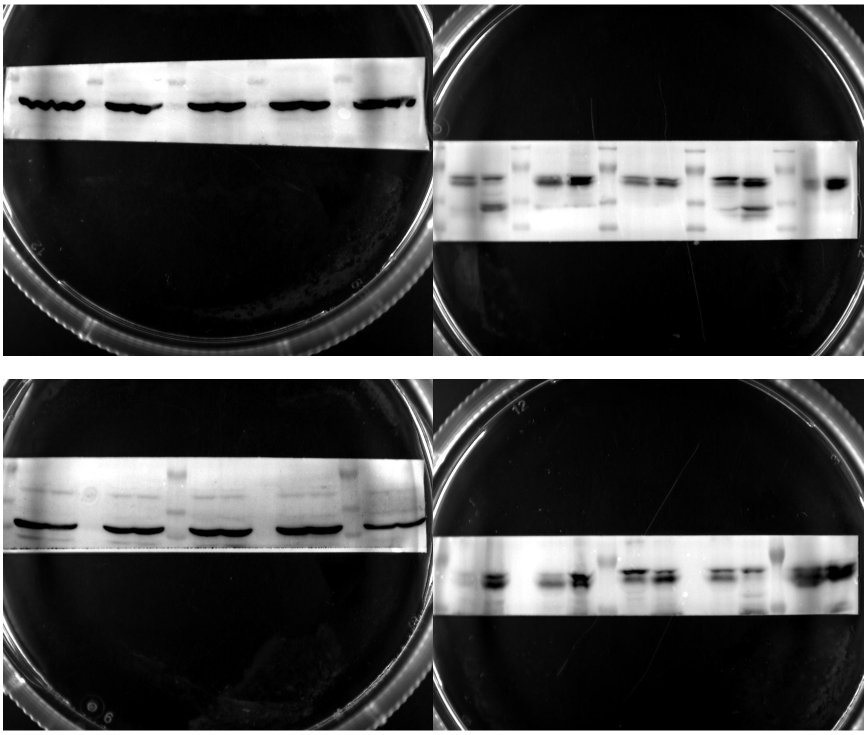

Fig 1F

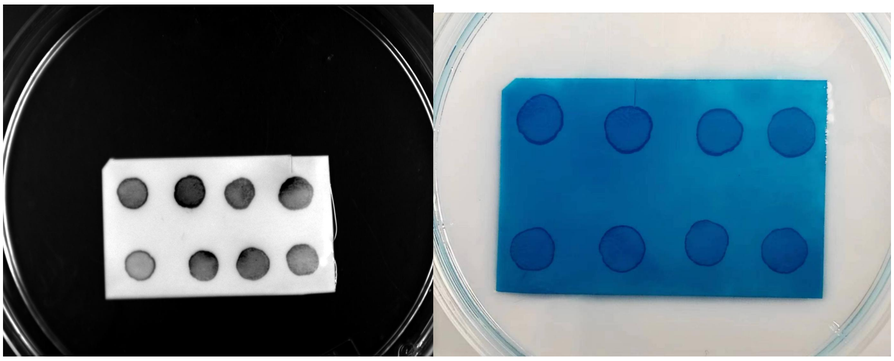

Fig 1G

Actin METTL3

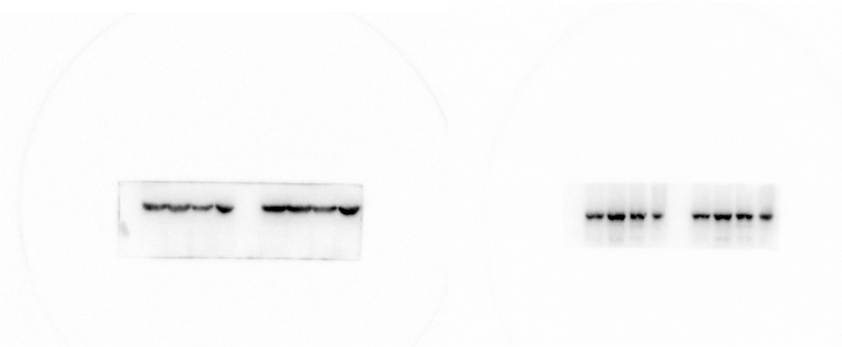

Fig 2A

Tubulin METTL3

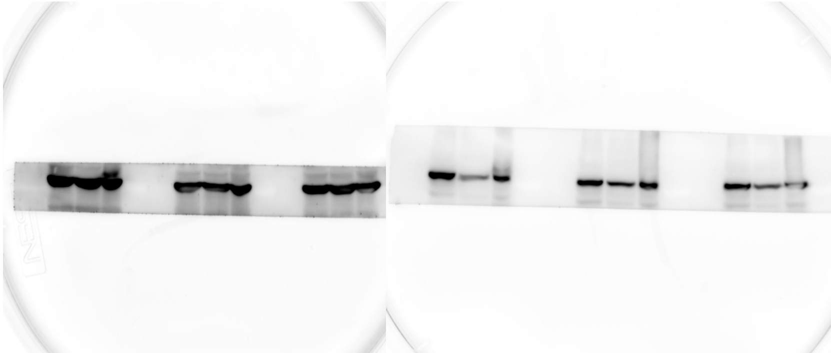

Fig 2B

METTL3 Tubulin

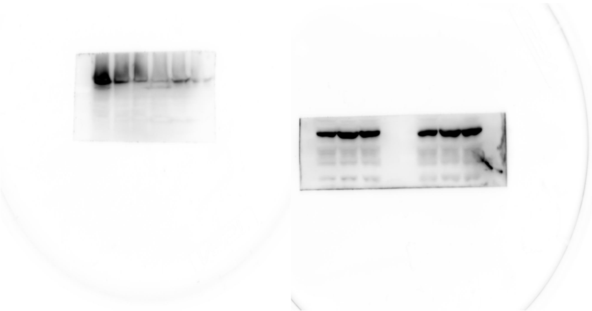

Fig 3A

Left

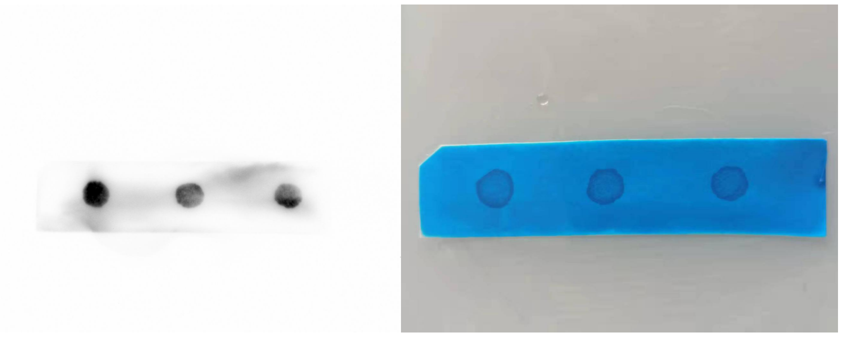

Right

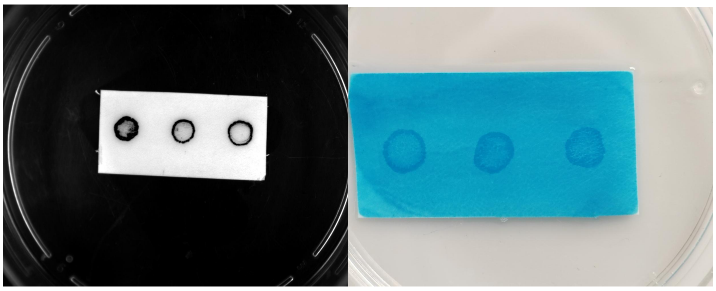

Fig 3D

Left METTL3

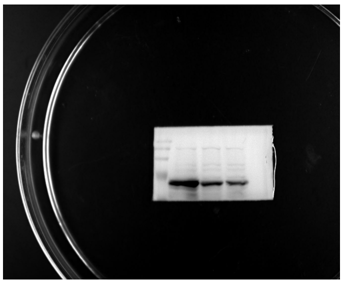

STAT3

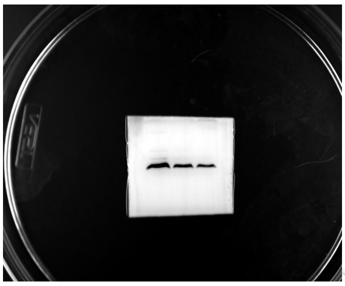

Actin

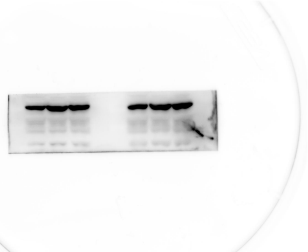

P-STAT3

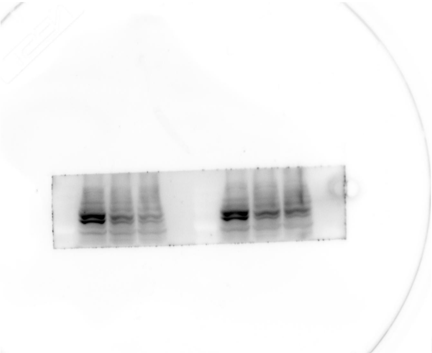

JAK1

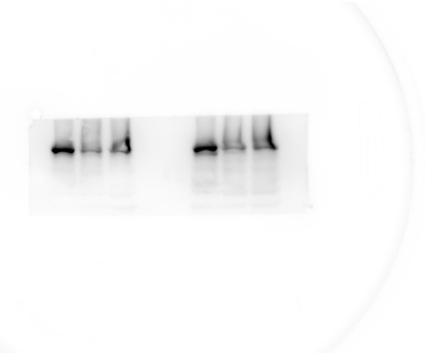

Right

Actin

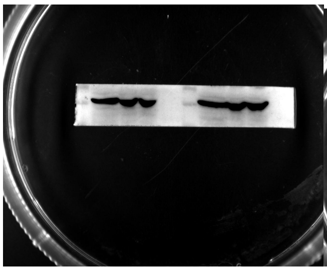

JAK1

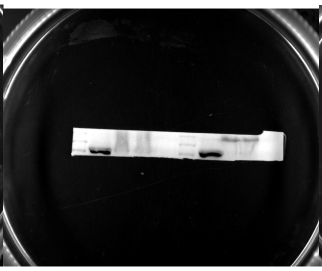

METTL3

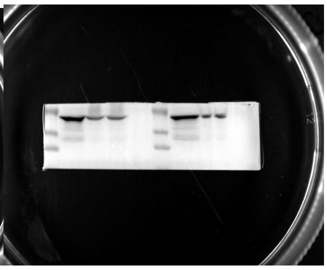

P-STAT3

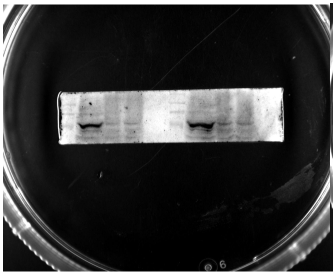

STAT3

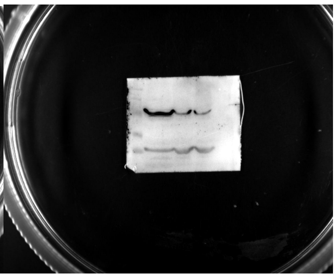

Fig 3F

METTL3

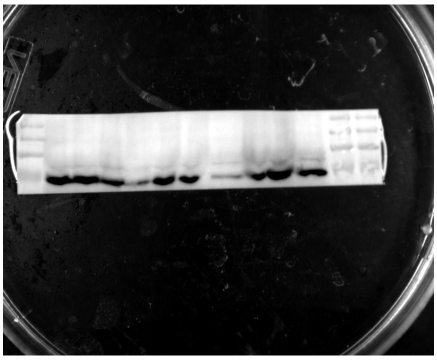

Tubulin

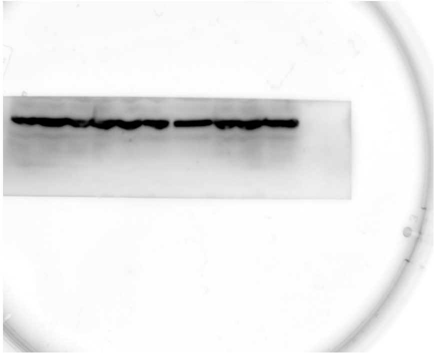

Fig 3G

JAK1

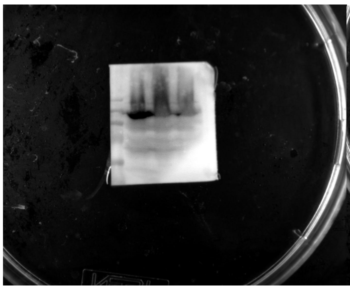

P-STAT3

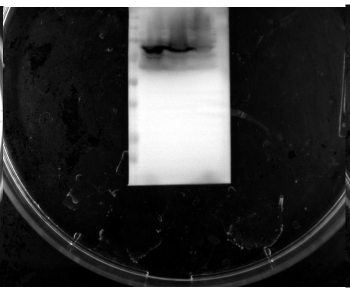

STAT3

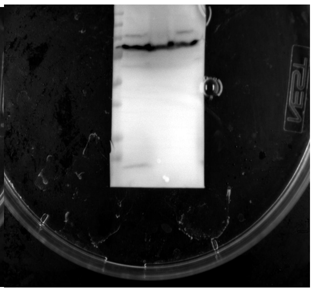

Actin

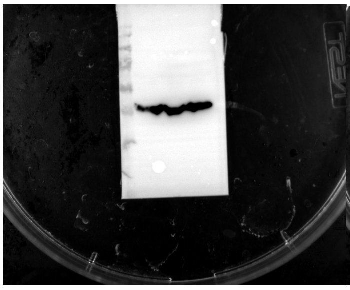

METTL3

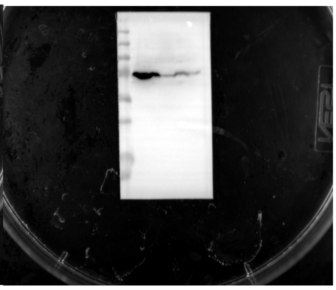

Fig 3I

METTL3

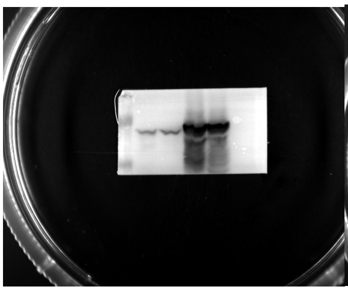

Actin

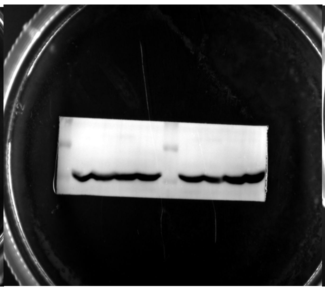

JAK1

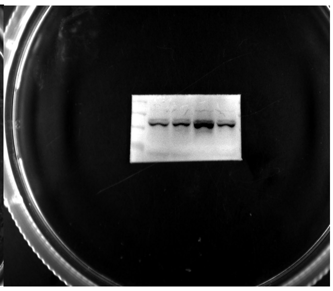

Flag

STAT3

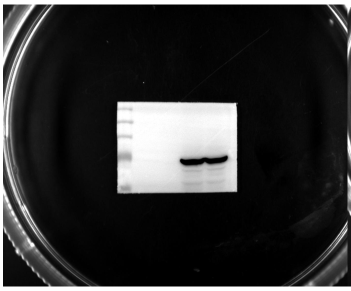
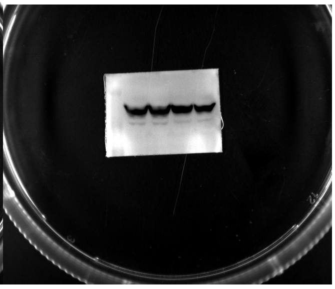

Fig 4A

Actin JAK1 METTL3

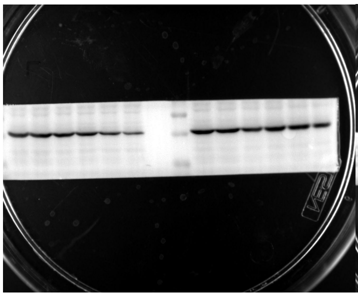
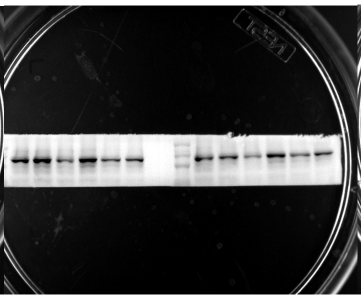
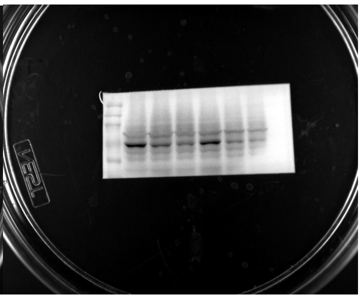

Fig 4C

Actin JAK1 P-STAT3

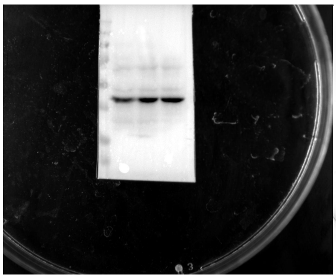
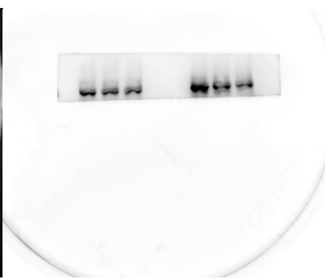
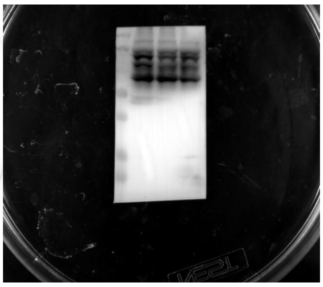

STAT3 YTHDF1

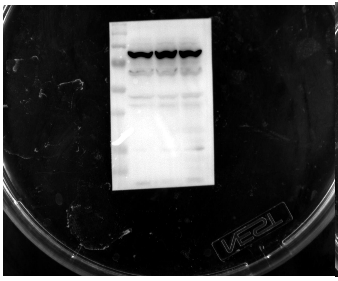
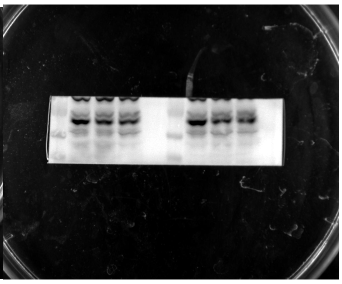

Fig 4D

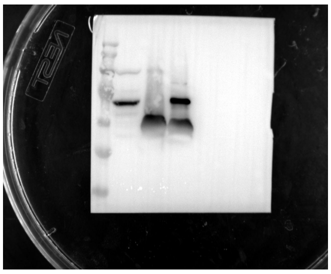

Fig 5D

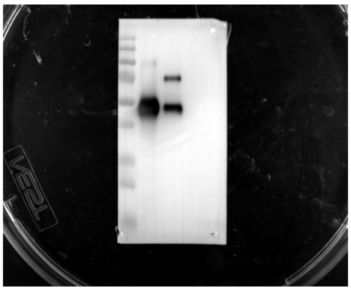

Fig 5E

NF-KB

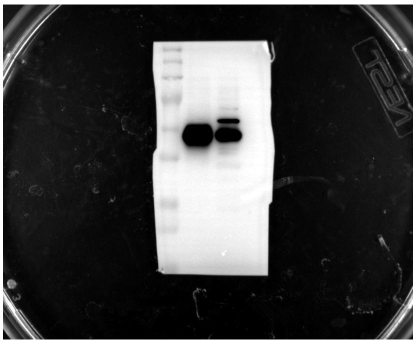

Fig 5F

METTL3 NF-KB Actin

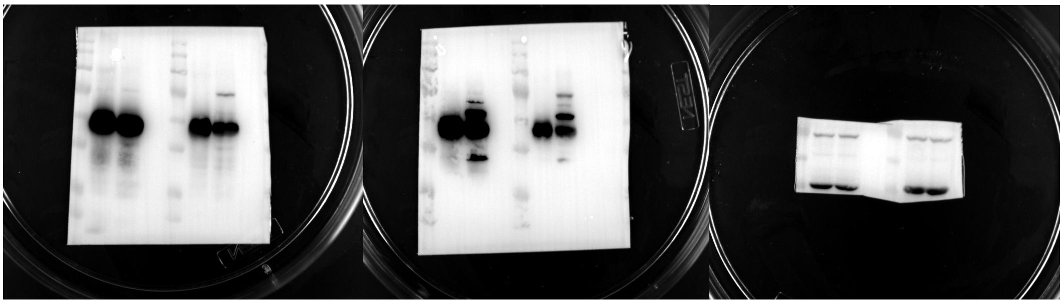

Fig 5H

Actin STAT3 NF-KB(P65)

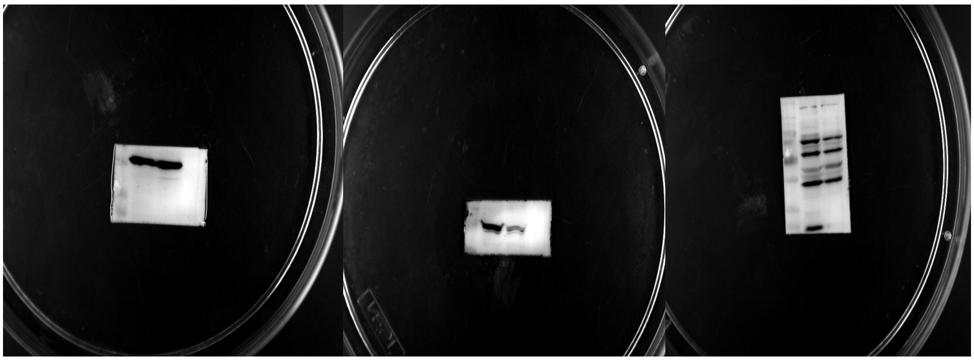

Fig 6B

Actin METTL3 VEGF

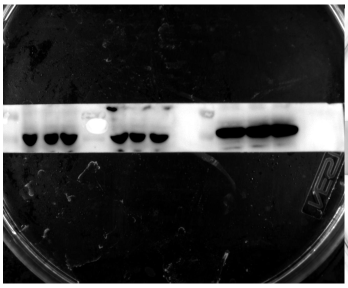
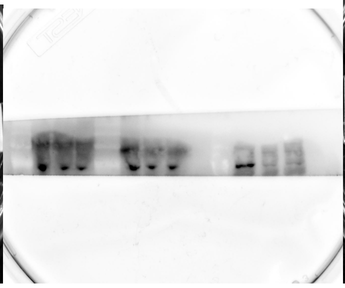
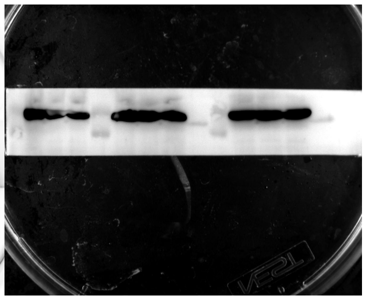

Fig 6C

CCND1 Actin VEGFA

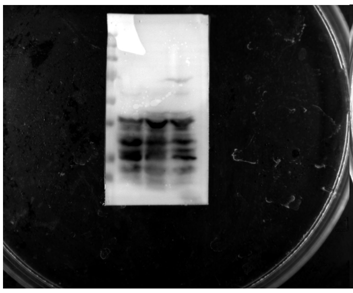
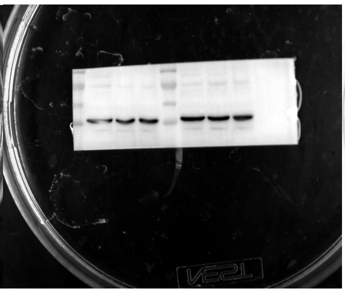
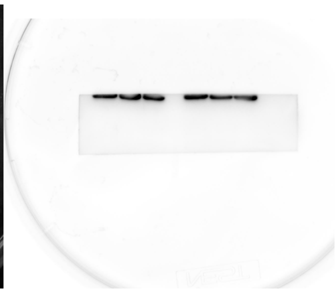

METTL3

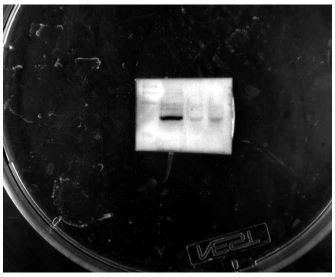

Fig 6D

VEGFA Actin CCND1

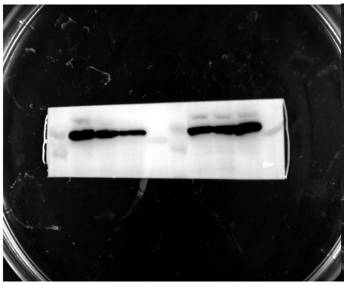
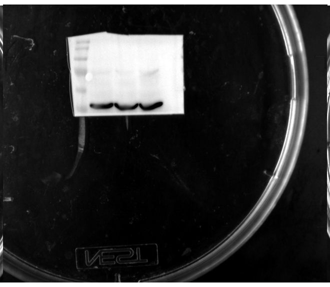
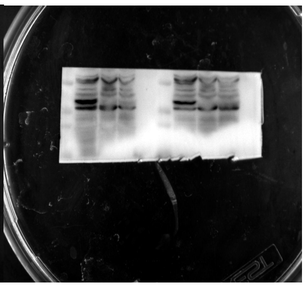

YTHDF1

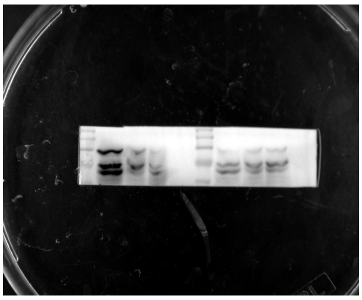

Fig 6E

CCND1 P-STAT3 Tubulin

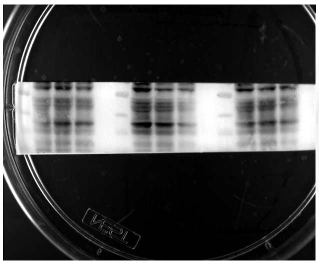

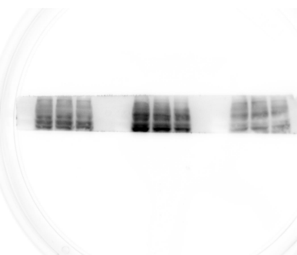

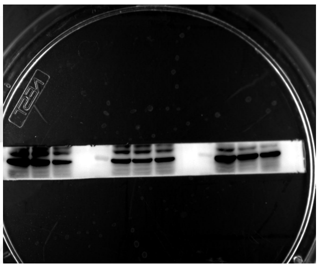

VEGFA STAT3

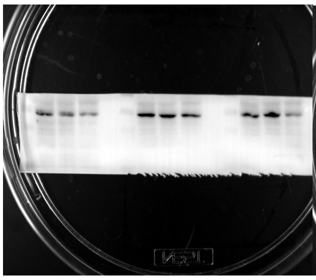

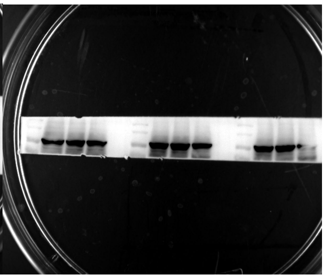

Fig 7K(left)

Actin METTL3 JAK1

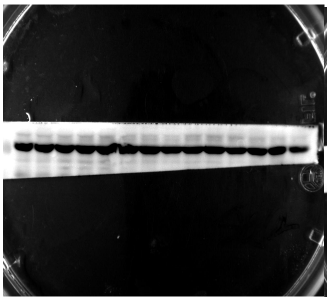

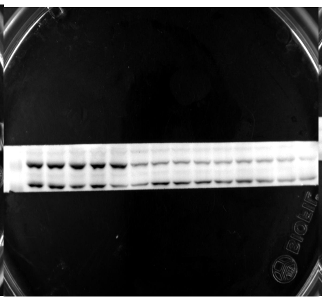

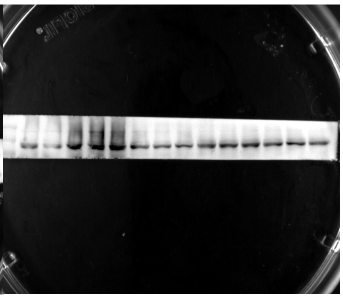

STAT3

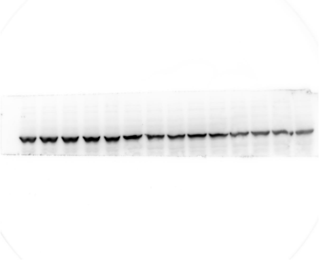

Fig 7K(right)

P-STAT3

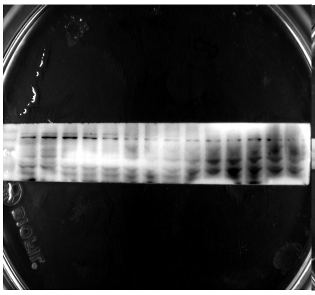

JAK1

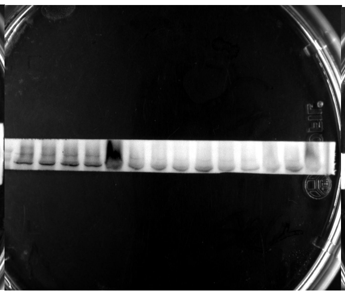

YTHDF1

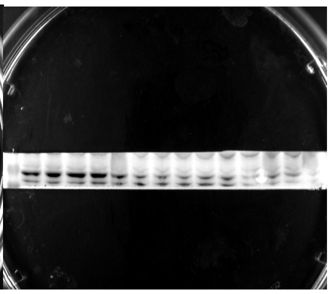

Actin

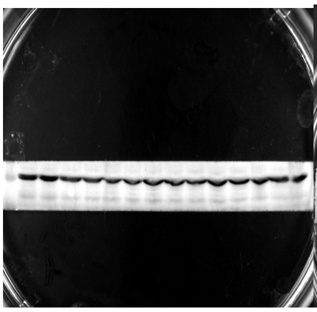

STAT3

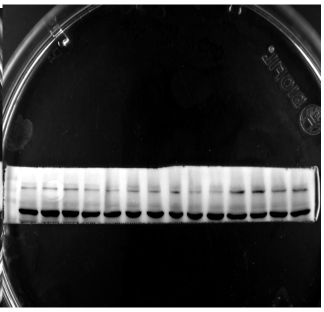

Supplement: Supplementary file 3 — Uncropped western blots [file 41419_2023_6287_MOESM3_ESM.pdf]
